# Digital Contingency Management for Substance Use Disorder Treatment: 12-Month Quasi-Experimental Design

**DOI:** 10.2196/73617

**Published:** 2025-09-02

**Authors:** Xiaoni Zhang, Valerie Hardcastle

**Affiliations:** 1 Department of Management, Information Systems and Quantitative Methods, The University of Alabama at Birmingham, CSB 371 710 13th Street South, Birmingham, AL, 35294, United States, 1 2059348890, 1 2059348830; 2 Institute for Health Innovation, Northern Kentucky University, Highland Heights, KY, 41099, USA

**Keywords:** digital contingency management, contingency management, substance use disorder, outcome, mobile app, abstinence, appointment attendance

## Abstract

**Background:**

Although contingency management has shown some efficacy in substance use disorder treatment, digital contingency management (DCM) needs more evidence supporting its value in treating substance misuse.

**Objective:**

This study aimed to evaluate the effectiveness of DCM in treating substance use disorder by examining 2 key outcome variables—abstinence and appointment attendance.

**Methods:**

A 12-month quasi-experimental design was conducted by enrolling patients into 2 groups using an alternating assignment process: one group receiving treatment-as-usual plus DCM and the other receiving treatment as usual with no contingency management. Propensity score matching was conducted to match groups on covariates. After matching, *t* tests were conducted to examine the difference between groups on urine abstinence and appointment attendance rates.

**Results:**

Two cohorts of propensity-matched patients (66 interventions and 59 controls) were analyzed. Abstinence was significantly higher in the DCM group (mean 0.92, 95% CI 0.88‐0.96) than in the treatment-as-usual group (mean 0.85, 95% CI 0.79‐0.90; *P*<.01). Appointment attendance also demonstrated significant differences between the groups, with the DCM group achieving a mean rate of 0.69 (95% CI 0.65‐0.74) compared with 0.50 (95% CI 0.45‐0.55) in the treatment-as-usual group (*P*<.001). This notable increase highlights the role of DCM in fostering engagement with care, an essential factor for successful treatment outcomes.

**Conclusions:**

The results suggest that DCM can be an effective treatment modality for substance use disorder.

## Introduction

### Background

Substance use disorders (SUDs) are a worldwide public health concern that impacts millions of people and weighs heavily on health care systems, families, and communities [1,2]. Over the past decade, substance use problems have been linked to a significant rise in both sickness and death rates globally [3]. Even with the presence of proven treatments, treatment-related attendance and follow-up remain significant barriers to recovery, and the rate of dropout ranges between 20% and 70% for different treatment programs [4,5]. Treatment adherence (ie, appointment attendance) and continued substance abstinence are 2 crucial elements that impact the outcome of treatment [6,7]. However, traditional treatment protocols struggle with multiple problems, such as low appointment attendance as well as long-term abstinence and relapse rates [8].

Contingency management (CM) is one of the most research-validated behavioral treatments to increase abstinence and treatment adherence in SUD treatment [9]. The foundation of CM is operant conditioning, which holds that actions leading to favorable outcomes are more likely to be repeated, whereas actions that result in unfavorable outcomes are less likely to be repeated. CM can be used to address several health behaviors, including medication adherence, substance use, weight loss, and quitting smoking. Based on behavioral economics and operant conditioning, this methodology is a systematic offering of material reward or incentive, depending on verified target outcomes such as passing drug tests or attending appointments [10]. The efficacy of CM has been consistently shown in meta-analyses for a range of SUDs, and in some studies, CM can boost medication adherence, therapy attendance, and abstinence rates [8]. Despite this solid evidence base, traditional CM implementation has limitations, including excessive administrative overheads, frequent in-person testing of target behavior, cumbersome reward systems, and policy obstacles, such as US federal antikickback and beneficiary inducement monetary and criminal sanctions [11].

### Previous Work

Digital contingency management (DCM) combines the traditional CM paradigm with digital health tools, which is the next generation of SUD treatment delivery. DCM involves using remote, technology-based techniques (such as the internet and smartphones) to track drug status using biochemical sensors (such as breath alcohol and carbon monoxide monitors) and to provide desired outcomes, such as monetary rewards depending on drug abstinence [12,13]. Such online enhancements are not only efficient at providing CM but also offer unparalleled data collection and program optimization.

Digital technologies offer multiple benefits for CM over other forms of implementation. First among these is greater accessibility as digital services transcend geographic boundaries and transportation expenses, especially for patients living in congested urban, rural, or underserved communities. Digital platforms remove much of the administrative cost as automated solutions cut staff time compared with manual CM systems while keeping similar verification accuracy rates. In addition, DCM systems’ real-time data collection capabilities allow dynamic program optimization: machine learning algorithms can recognize patterns in patient usage and adjust reward schedules to optimize treatment. Data show that personalized digital incentives better drive program retention than traditional fixed-schedule solutions [14]. DCM implementations may be affordable, with programs averaging per-patient costs less than traditional CM while achieving equal or better outcomes.

Even with the promising potential of digital CM, there is still a lack of comprehensive understanding of how it compares with traditional CM methods. Existing literature provides substantial evidence supporting the efficacy of traditional CM in SUD treatments, particularly for smoking cessation and alcohol use disorders [15]. However, more research is needed to evaluate its effectiveness in treating drug use. While DCM shows significant potential as an innovative approach, there remains a critical need for robust empirical evidence to validate its efficacy and broader applicability [15].

### Study Objectives

CM is underutilized in clinical settings [12]. If proven effective, DCM could present a better modality for widespread adoption. There is a pressing need to understand the interrelationships between appointment attendance, abstinence outcomes, and DCM interventions [10,16]. This understanding is crucial for developing more effective, integrated treatment approaches to better support individuals in their recovery journey [17].

In this study, we attempted to fill critical gaps in understanding the impact of DCM on SUD treatment by evaluating its effectiveness on 2 key outcomes: appointment attendance and abstinence. In particular, we compared a standardized DCM intervention with treatment as usual across multiple sites in a time-randomized controlled trial. These results will be part of the scientific evidence base for digital behavioral interventions in SUD treatment and should guide the development of more effective, accessible, and long-term treatment solutions. Here, we aim to provide some evidence-based recommendations for enhancing SUD treatment protocols. This research aimed to answer the following research questions: (1) How does DCM impact treatment adherence, as measured by appointment attendance? (2) How does DCM impact treatment outcomes, as measured by substance test abstinence rates?

## Methods

### Study Design

DynamiCare Health provided the DCM platform. We conducted a 12-month quasi-experimental study of DCM in a SUD population seeking treatment in partnership with providers located in the Greater Cincinnati Metropolitan Statistical Area. This quasi-experimental design used 2 groups to compare treatment outcomes between a non-DCM treatment-as-usual control group and a DCM experimental group. Participants were assigned to their group using an alternating assignment process based on the sequence of their enrollment in the program, rather than through random assignment. This approach was adopted due to pragmatic and ethical constraints in operating in a real-world clinical setting. Pure randomization was not feasible at intake because clinicians had to obtain informed consent before assigning a patient to the experimental group. To mitigate bias, intake coordinators were instructed to follow a strict alternating pattern, regardless of patient characteristics or interest, with no discretion in who was offered participation.

The study was carried out over a 4-year period from 2021 to 2024. Clients used the DynamiCare Health mobile app on smartphones to check into their treatment appointments (verified by GPS) and track any financial rewards they earned. These rewards were provided on a smart debit card that blocked access to charges in bars, liquor stores, and cash withdrawals.

Due to practical constraints of this real-world implementation study, a formal a priori power analysis was not conducted. The sample size was determined by the number of eligible patients who consented to participate in the study during the enrollment period. While our final matched sample (66 interventions and 59 controls) was relatively modest for a 12-month study, it proved sufficient to detect statistically significant differences in our primary outcomes. However, we acknowledge that the relatively small sample size may have limited our ability to detect smaller effect sizes, particularly for secondary outcomes or subgroup analyses.

In our study, missing data and attrition were minimal. Participants’ appointment attendance and urine test results were extracted from electronic health records, which systematically document all scheduled appointments and urine screens, including missed visits and missed tests. Therefore, nonattendance and missing urine tests were captured as part of the outcome measurement rather than treated as missing data.

Participants who disengaged from treatment or failed to provide urine samples were included in the denominator when calculating appointment attendance rates and urine test success rates, in accordance with an intention-to-treat principle. No participants were excluded due to missing data. As a result, there was no attrition bias requiring adjustment.

### Procedure

Patients were assigned to groups using an alternating assignment process. During intake, an intake coordinator described the DCM project to the first patient. If this patient were interested in participating, they were consented and placed in the experimental group. The next patient the intake coordinator saw would automatically be placed in the treatment-as-usual control group without receiving any project description. This alternating process continued, offering participation in the project to every other patient. The intake coordinator then informed therapists of each patient’s group assignment, and therapists explained the DynamiCare system to those in the experimental group during their second appointment. Participants in the control group were not given any further instructions; they just received treatment as usual. The electronic health record system documented all treatment events in which the experimental and control groups participated and their medical histories.

The enrollment order assignment is not true randomization and could introduce bias. To address this, we used patient ID as a proxy for enrollment sequence and tested whether our outcomes showed systematic trends over the enrollment period. To address this, we conducted time series analyses to check for secular trends in our outcome measures during the enrollment period. We found no significant temporal patterns in either abstinence rates (*P*=.59) or appointment attendance (*P*=.47) across the study time frame for the experimental group. We conducted the same analyses for the control group with similarly nonsignificant results (*P*=.49 for urine abstinence rate and *P*=.28 for appointment rate). These findings provide strong evidence that time-dependent confounding was not a concern in our study. The lack of significant temporal trends in our outcomes suggests that the sequential assignment of participants did not introduce systematic bias beyond what was addressed by our propensity score matching approach.

### Intervention DCM

There were only 2 differences between the experimental group and the control group. First, the experimental group received financial awards for completing prescribed treatment activities and for remaining sober. The control group engaged in the same treatment and testing regimen; however, it received no financial rewards for success. And second, the experimental group used a phone app as part of its treatment protocol, while the control group did not use a phone app as part of its treatment protocol. The experimental group’s app allowed the participants to check into their appointments online, which DynamiCare could then use to verify attendance by a location tracker. The app also tracked the cash value to the experimental group participants’ debit card as they received rewards for complying with their prescribed treatment activities and purchased allowable items or services. The DynamiCare company disbursed the reward funds as each of the experimental participants completed each prescribed activity successfully.

Patients in the experimental group used their own iOS or Android smartphones in this study. The intake coordinator assisted patients in downloading and logging into the app for the first time. At the time of primary data collection for this study, the HIPAA (Health Insurance Portability and Accountability Act)-compliant mobile DynamiCare app was used only to locate patients during scheduled therapeutic appointments and to reward patients completing specific, verified, and scheduled activities (the app now includes many interactive features).

In the experimental group, cash rewards were promptly provided for attending scheduled counseling appointments and receiving a negative outcome in urine screens. The system recorded negative “weeks since onboarded” as an automatic process from the account creation. Usually, patients downloaded the app shortly after receiving the invitation, but there were occasions when the users did not access the app for weeks (or at all). Four sites implemented the intervention between October 16, 2020, and September 13, 2023.

Participants could earn up to US $600 in rewards over a 12-month period, with incentives front-loaded during the first 3 months to foster engagement and a pattern of active effort. During this initial period, participants could earn US $100 per month, with the rewards tapering down in subsequent months. Specifically, months 4‐6 offered US $50 per month, months 7‐9 provided US $40 per month, and months 10‐12 offered US $25 per month.

Both experimental and control group participants underwent the same therapy and urine testing sequences, following the protocols of their individual behavioral health units. Both the experimental and control groups participated in clinical activities, as per the protocols of the individual treatment facilities (these did not differ by group, although they could differ by individual, depending on medical recommendations). But the recommendations were not determined by app usage; rather, in both groups, participants were treated as they ordinarily would have been, regardless of the DCM intervention.

### Outcome Variables

The outcome variables were abstinence and appointment attendance. Abstinence was measured using objective methods, specifically, urine drug screening. Urine test success is measured as the number of negative urine test results or medically consistent results divided by the total number of urine tests logged by providers in their electronic medical records. A test was marked consistent, or abstinent, if it met medical expectations: negative for illicit substances, with positive results for prescribed substances allowed. Appointment attendance was calculated as the number of attended appointments divided by the total number of scheduled appointments.

All participants who enrolled in the program were included in the analysis regardless of their level of engagement. Missed appointments and missing urine screens were treated as part of the outcome definitions rather than missing data, using the following formula for appointment attendance rate: appointment attendance/(total number of missed appointments + total number of attended appointments). No participants were excluded due to missing information, ensuring a complete analysis under an intention-to-treat framework. We examined the potential time-dependent confound arising from our enrollment order assignment and did not find any significant temporal trends in our outcomes.

While we did not conduct a formal a priori power analysis due to the pragmatic nature of this implementation study, we assessed the adequacy of the sample size post hoc by evaluating the observed effect sizes and associated *P* values for our primary outcomes. We used G*Power (version 3.1.9.7) to determine the statistical power of the study. To detect a medium effect size (Cohen *d*=0.50) with a 1-tailed independent samples *t* test at a significance level of α=.05, and using our matched sample sizes (66 in the treatment group and 59 in the control group), we achieved a power of 87.1%. This means that there is an 87.1% probability of detecting a true medium-sized effect if one exists, which exceeds the conventional 80% threshold typically considered acceptable in most fields. Our matched sample of 125 participants (66 treatment and 59 control) thus provides sufficient power to detect medium effects in our primary outcomes. We have added this explanation to the “Study Design” section.

### Propensity Score Matching

Propensity score matching (PSM) was used to remove confounding bias [18-22] as our experimental study was randomized based only on the time of program entry. To control for selection bias, we used nearest neighbor 1:1 matching. A binary logistic regression was used to estimate the propensity score. Variables’ association with treatment and outcome, as well as both treatment and outcome, was considered and reported. The variables used in the PSM model included gender, race, relationship status, and insurance. Selection of these covariates was based on previous research [23] reporting on the impact that various sociodemographic factors have on appointment attendance and substance use outcomes among individuals receiving treatment for SUDs. In particular, women are less likely to receive SUD treatment than men [24]. Marriage was associated with lower rates of SUD [25]. Race and ethnicity have been linked to treatment completion [26]. Health insurance coverage seems to be linked to better abstinence outcomes among US adults with mental health and substance use disorders [27].

This study aimed to estimate the effect of DCM on abstinence and appointment attendance. The challenge in estimating these effects is to plausibly separate them from confounders that might affect patients’ outcomes and to isolate those associated with digital CM. PSM was performed to design balanced samples. We selected an experimental group for each patient receiving DCM that had similar observed characteristics at intake. PSM was performed using a 1:1 nearest neighbor matching algorithm with replacement with distances determined by logistic regression using the Stata package PSMatch2. Standardized mean differences (SMDs) were used to evaluate the balance between treated patients and matched controls. Before and following matching, this measure is used to balance the variables [18]. SMDs less than 0.2 suggested a reasonable balance for group comparison following matching [21]. After matching, standardized differences between covariates were generally well balanced with values ranging from 0.01 to 0.08, within the recommended threshold of less than 0.2. However, gender maintained a moderate imbalance with an SMD of 0.23, slightly exceeding our predefined threshold. This residual imbalance was considered in subsequent analyses and interpretation.

To assess the quality of matching, SMDs were calculated and checked for all variables included in the propensity score model. Following Austin’s recommendation, we considered SMDs less than 0.2 to indicate adequate balance between treated and control groups. To evaluate the robustness of our findings to potential unmeasured confounding, we conducted postmatching sensitivity analysis using Rosenbaum bounds. This approach helped determine how significant an unmeasured confounder would need to be to negate the observed treatment effects. The sensitivity analysis tested a range of gamma values (Γ) from 1 to 3, with Γ=1 representing no hidden bias and higher values indicating increasingly strong unmeasured confounding.

### Statistical Analysis

Group comparison and linear regression were used to assess the average treatment effect of DCM on abstinence and appointment adherence. Statistical analyses were performed in Stata (version 18; StataCorp) and 2-sided *P* value of <.05 indicated statistical significance. *P* values for continuous values were calculated using a weighted *t* test, and a chi-square test was used for categorical comparisons. We also conducted the robustness check by conducting a sensitivity analysis.

### Ethical Considerations

Institutional review board (IRB) approval was obtained from the Northern Kentucky University IRB (IRB number 914) under expedited review according to 45 CFR 46.110: (4) collection of data through noninvasive procedures and (7) research on individual or group characteristics or behavior. Participant consent was obtained at the treatment facilities during patient onboarding; all possible participants could opt out of the study at any time. Consent language included: “Your participation is completely voluntary; you are free to change your mind at any time and quit the study. Whatever you decide will in no way result in loss of medical or health benefits or services to which you are otherwise entitled.”

There were no secondary analyses using data with primary consent. All patient information and data were deidentified using random numbers as identifiers. No one engaged in data analysis had access to any identifying information. All data associated with this study are kept on a secure HIPAA-compliant server. Because participant compensation comprises part of the study design, it is described in the “Intervention DCM” subsection.

## Results

### Overview

**Figure 1.**
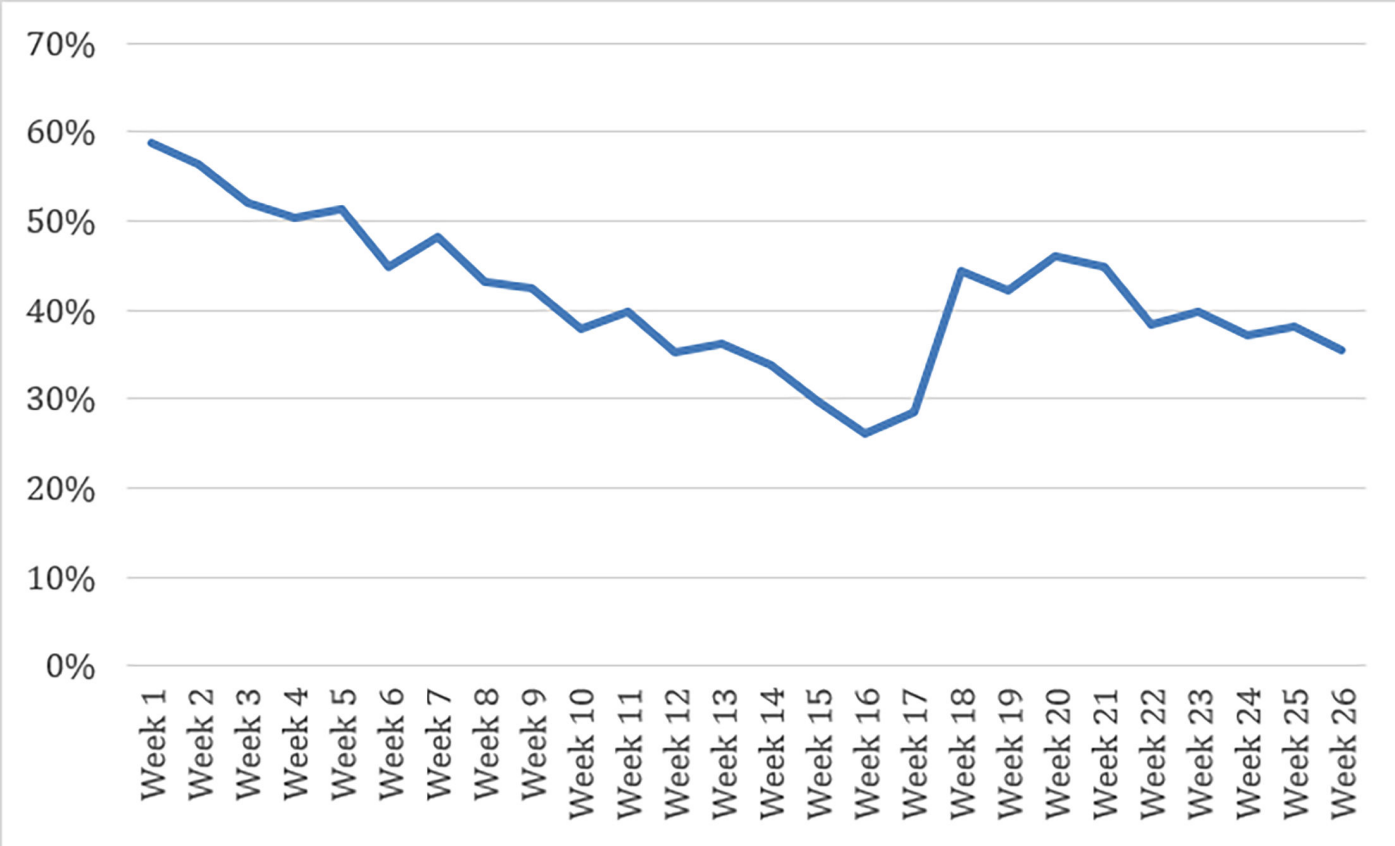
Urine consistency rate with digital contingency management.

Figures1  2 show the consistent urine rate and appointment attendance rate for patients using DCM.

**Figure 2.**
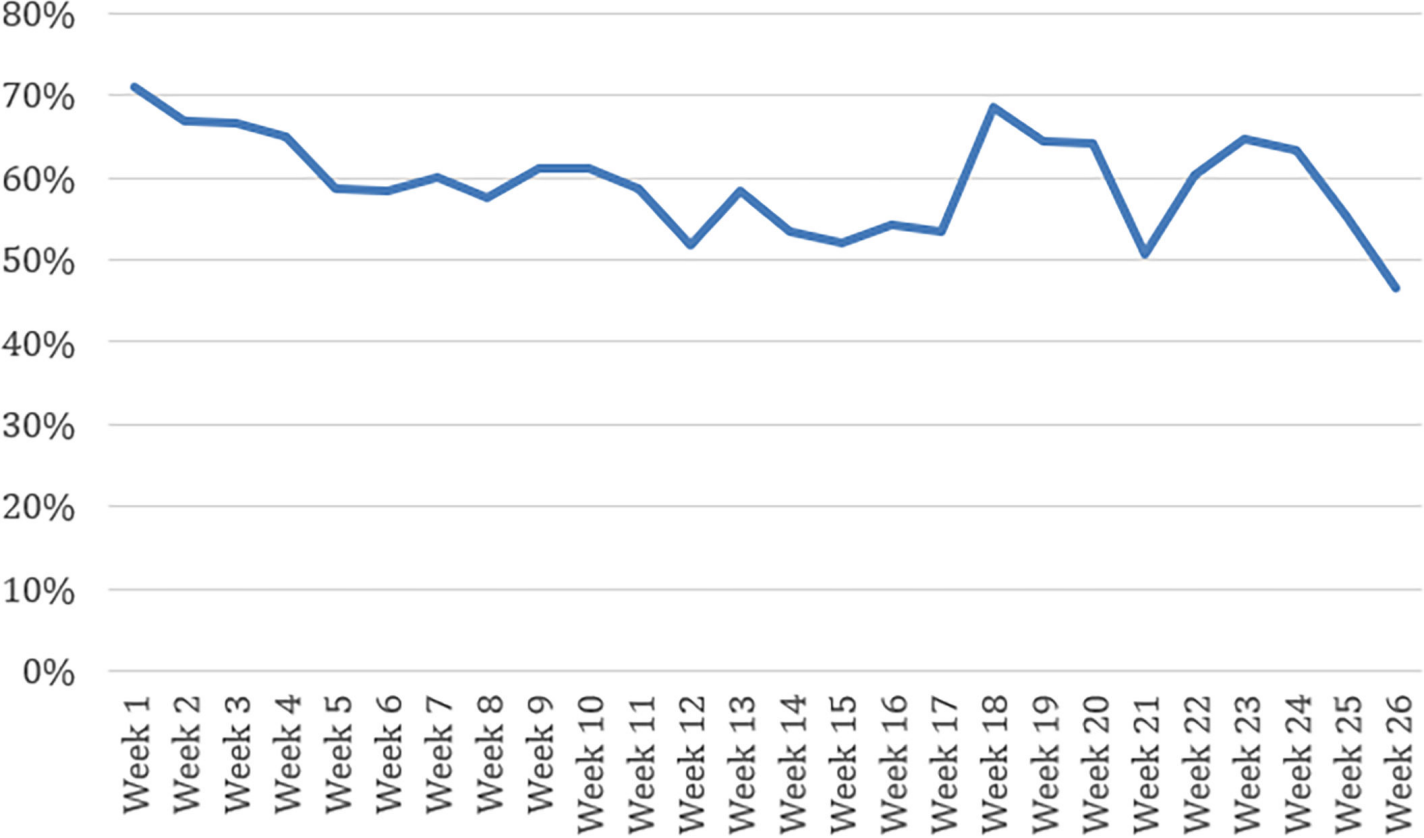
Appointment attendance rate with digital contingency management.

### Cohort Characteristics

Table 1 shows the demographic and insurance-related characteristics of the experimental group (n=74) and the control group (n=119) in the unmatched sample. The *P* values indicate statistical significance, and standardized differences provide a measure of the imbalance between groups. Females were more prevalent in the experimental group (44/74, 59.5%) than in the control group (30/74, 42.86%). Males were more prevalent in the control group (68/119, 57.14%) than in the experimental group (30/74, 40.5%). With *P*=.025 and SMD=−0.31, there is a significant difference between groups in terms of gender. Race and education exhibit comparable characteristics between the unmatched cohorts. Relationship status shows a moderate imbalance (*P*<.05; SMD=−0.58), while insurance type shows a significant imbalance (*P*<.02; SMD=0.35).

**Table 1.** Comparison of characteristics of study cohorts in the original unmatched sample.

Characteristics	Treatment (n=74)	Control (n=119)	*P* value	Standardized differences
Sex, n (%)			.03	−0.31
Female	44 (59.50)	51 (42.86)		
Male	30 (40.50)	68 (57.14)		
Race, n (%)			.37	0.11
Black	16 (21.62)	N/A^a^		
Hispanic or Latino	2 (2.7)	20 (16.8)		
Other race	2 (2.7)	3 (2.5)		
White	54 (72.97)	96 (80.7)		
Education, n (%)			.74	0.02
Associate’s degree	3 (4.05)	3 (11.11)		
Bachelor’s degree	2 (2.70)	1 (3.7)		
High school diploma	28 (37.84)	7 (25.93)		
Less than high school	19 (25.68)	6 (22.22)		
Master’s degree	1 (1.35)	0 (0.00)		
Some college	16 (21.62)	8 (29.63)		
Unknown	5 (6.76)	94 (78.99)		
Relationship, n (%)			.05	−0.58
Divorced	7 (9.46)	5 (4.2)		
In a relationship	4 (5.41)	0 (0.00)		
Married	11 (14.86)	7 (5.88)		
Single	50 (67.57)	38 (31.93)		
Unknown	2 (2.7)	64 (53.78)		
Separated	N/A	5 (4.2)		
Insurance^b^, n (%)			.02	0.35
Commercial	5 (6.76)	21 (17.65)		
Medicaid	58 (78.38)	50 (42.02)		
Medicare	9 (12.16)	11 (9.24)		
Not insured	2 (2.7)	4 (3.36)		
Medicaid and Medicare,	N/A	1/119 (0.84)		
Urine rate, mean (95% CI)	0.92 (0.77-1)	0.86 (0.71-1.0)	N/A	0.34
Appt rate, mean (95% CI)	0.65 (0.44-0.87)	0.62 (0.39-0.84)	N/A	0.43

a N/A: not applicable.

b This is insured by both Medicare and Medicaid.

Table 2 shows the characteristics of matched cohorts. After matching, the sample size for the treatment was 66, whereas the sample size for the control was 59. All the characteristics are well balanced between the matched cohorts. While most variables achieved excellent balance after matching (SMDs <0.1), gender retained a moderate imbalance (SMD=0.23) despite the matching procedure. This moderate imbalance in gender distribution represents a limitation of our matching process that warrants consideration when interpreting the results.

**Table 2.** Comparison of characteristics between treatment and control participants in the propensity score matched sample.

Characteristics	Treatment (n=66)	Control (n=59)	*P* value	Standardized difference
Sex, n (%)			.44	0.23
Female	37 (56.06)	29 (49.15)		
Male	29 (43.94)	30 (50.85)		
Race, n (%)			.40	0.02
Black	14 (21.21)	16 (27.12)		
Hispanic or Latino	2 (3.03)	0 (0.00)		
Other race	1 (1.52)	3 (5.1)		
White	49 (74.24)	40 (67.8)		
Education, n (%)			.79	0.06
Associate’s degree	3 (4.55)	3 (11.11)		
Bachelor’s degree	2 (3.03)	1 (3.7)		
High school diploma/GED	25 (37.88)	7 (25.93)		
Less than high school	16 (24.24)	6 (22.22)		
Master’s degree	1 (1.52)	0 (0.00)		
Some college	14 (21.21)	8 (29.63)		
Unknown	5 (7.58)	2 (7.41)		
Relationship, n (%)			.15	0.08
Divorced	6 (9.09)	5 (8.47)		
In a relationship	1 (1.52)	0 (0.00)		
Married	11 (16.67)	7 (11.86)		
Single	46 (69.7)	38 (64.41)		
Unknown	2 (3.03)	4 (6.78)		
Separated	0 (0.00)	5 (8.47)		
Insurance, n (%)			.35	0.03
Commercial	5 (7.58)	4 (14.81)		
Medicaid	50 (75.76)	21 (77.78)		
Medicare	9 (13.64)	2 (7.41)		
Not insured	2 (3.03)	N/A^a^		
Urine rate, mean (95% CI)	0.92 (0.76-1.09)	0.84 (0.64-1.05)	N/A	0.34
Appointment rate, mean (95% CI)	0.69 (0.5-0.89)	0.49 (0.3-0.69)	N/A	0.43

a N/A: not applicable.

### Abstinence

After PSM, we conducted *t* tests to compare urine rate between the control and experimental groups for the matched sample of 125 observations (59 controls+ 66 treated). Table 3 shows the urine test consistency rate test statistics between the treatment and control cohorts. This suggests that the treatment intervention likely had a positive impact on urine rate outcomes. The urine test abstinence rate, as indicated by the urine rate, was significantly higher in the DCM group (mean 0.92, 95% CI 0.88‐0.96) than in the treatment-as-usual group (mean 0.85, 95% CI 0.79‐0.90; *P*<.01).

**Table 3.** *t* test results after matching.

Group	Observed	Mean (SD)	95% CI	*P* value
Urine rate				.01
Control	59	0.85 (0.21)	0.79-0.90	
Treatment	66	0.92 (0.16)	0.88-0.96	
Appointment rate				.001
Control	59	0.50 (0.20)	0.45-0.55	
Treatment	66	0.69 (0.19)	0.65-0.74	

### Appointment Attendance Rate

Table 3 shows the appointment attendance rate statistics between the treatment and control cohorts. The appointment adherence rate was substantially higher in the DCM group (mean 0.69, 95% CI 0.65‐0.74) than in the treatment-as-usual group (mean 0.50, 95% CI 0.45‐0.55), with an even stronger statistical significance (*P*<.001).

### Sensitivity Analysis

We conducted a sensitivity analysis using Stata’s mhbounds command, which implements Rosenbaum bounds to evaluate the robustness of treatment effects to potential unmeasured confounding. This method assesses how much hidden bias (Γ) would be required to invalidate the statistical significance of the observed treatment effect. Our findings demonstrate that the treatment effects for both urine test rate and appointment attendance rate are robust to unmeasured confounding across a wide range of Γ values (1-3).

Complete sensitivity analysis results are shown in Tables S1 and S2 in Multimedia Appendix 1. Table S1 in Multimedia Appendix 1 on urine test rate shows that both sig+ and sig− values remain at 0 across all Γ values (1-2), indicating that the treatment effect maintains statistical significance even under strong assumptions of unmeasured confounding. The treatment effect estimate (t-hat) shows remarkable stability, ranging from 0.90 at Γ=1 to 0.86 (t-hat+) and 0.93 (t-hat−) at Γ=2. This minimal change suggests that the effect is highly robust. The CI values remain relatively narrow throughout (0.88‐0.91 at Γ=1 to 0.84-0.95 at Γ=2), further supporting the stability of the findings.

Table S2 in Multimedia Appendix 1 on appointment rate shows that both sig+ and sig− values remain at 0 across all Γ values, indicating sustained statistical significance. The treatment effect ranges from 0.67 at Γ=1 to 0.58-0.74 at Γ=2, showing slightly more variation than the urine rate analysis but still maintaining clinical significance. While the CI values widen somewhat with increasing Γ values (0.63‐0.69 at Γ=1 to 0.55-0.77 at Γ=2), they remain within interpretable bounds.

For both outcomes, the experimental group consistently outperformed the control group with statistically significant differences (*P*<.05). The minimal changes in treatment effect estimates and CI values across increasing levels of Γ suggest that our results are highly robust to potential unmeasured confounding. These findings support the conclusion that the intervention was likely effective in improving both urine test abstinence and appointment attendance rates.

## Discussion

### Principal Findings

Our study aimed to evaluate the effectiveness of DCM in comparison with treatment-as-usual for individuals with SUD. The findings reveal statistically significant differences in both urine test abstinence and appointment attendance among participants in the DCM group compared with the treatment-as-usual group. Patients in the DCM group showed higher urine abstinence rates (92% vs 85%; *P*<.01) and substantially better appointment attendance rates (69% vs 50%; *P*<.001). These results align with a previous study [28], which found that digital health interventions can effectively enhance medication adherence among individuals with opioid use disorder. Also, DeFulio [14] found that inner-city patients with SUD who were mostly treated with buprenorphine and the DynamiCare app showed significantly higher rates of treatment attendance in study months 2-4 (9.6%‐18.0% increases) and odds ratio of 4.84 for greater drug abstinence and medication adherence than the treatment-as-usual group (*P*<.05). These results highlight the potential of DCM as an innovative intervention for improving key treatment outcomes in SUD care. Our findings extend the existing literature on traditional CM approaches and contribute to the much-needed evidence on the efficacy of DCM for drug treatment, supporting the broader transition toward digital therapeutic interventions in addiction treatment [12].

These results suggest that integrating digital tools into CM can enhance both treatment abstinence and engagement in care, consistent with previous studies highlighting the promise of digital interventions in substance misuse treatment [10,14]. This finding suggests that DCM interventions are effective in promoting abstinence, a critical metric in SUD treatment. Such gains align with previous findings on CM’s efficacy in motivating behavior change. Mobile apps can better communicate between the care team and the patients and could potentially save lives [29].

A 19% increase in appointment attendance with DCM is clinically meaningful, consistent with previous findings on digital interventions on treatment adherence [25]. This increase highlights the role of DCM in fostering engagement with care, an essential factor for successful treatment outcomes. By leveraging digital tools to deliver incentives, DCM may reduce the impact of logistical challenges, thus promoting consistent participation in scheduled appointments [30]. Compared with patients who received treat-as-usual alone, those who had treatment-as-usual coupled with app-based CM had a noticeably higher chance of remaining in treatment for longer [31]. These results corroborate earlier research that concluded that receiving rewards motivates patients to maintain treatment compliance [10].

The digital platform can decrease obstacles to incentive receipt and help remove infrastructure barriers that may impact attendance rates. Urban-rural disparities exist in CM availability [32]. Digital interventions could expand access to a broader patient population [33,34], suggesting that DCM could help reduce this disparity. Thus, DCM could help address the inequalities in health care support for the underserved groups [35,36].

According to earlier studies, digital interventions decrease the impact of logistical obstacles to participation, such as schedule conflicts and travel time, which are frequent problems in conventional therapy settings. The results support these views, which highlight DCM’s capacity to encourage long-lasting behavioral improvements in SUD populations [14,37]. In sum, DCM interventions align with behavioral reinforcement principles by providing timely rewards for desirable behaviors, thus enhancing adherence and reducing substance misuse [10].

### Limitations

Despite these promising findings, certain limitations warrant consideration. First, the study did not explore differences in outcomes across social determinants of health, motivation levels, or comorbidity. Future studies will explore the efficacy of DCM on SUD treatment relative to educational level, economic stability, transportation availability, and housing stability. Nevertheless, our sample’s high proportion of Medicaid and noncommercial insureds suggests that patients at risk due to social determinants of health challenges may benefit. The gender imbalance may affect the generalizability of our findings. Future research should include larger samples, powered to support more robust gender-stratified analyses.

Second, the study may have excluded individuals less familiar with digital technology. App features and user experience could affect engagement with DCM. This digital divide may disproportionately affect certain demographic groups, such as older adults or those from lower socioeconomic or educational backgrounds, potentially limiting the generalizability of the findings to all individuals with SUD. However, the front-loading of rewards is designed to motivate patients to overcome tech unfamiliarity.

Third, the study focused on outcomes, specifically drug abstinence and appointment adherence, during the intervention. While these measures are critical indicators of treatment success, the study did not assess the sustainability of behavior changes following the conclusion of the intervention. Follow-up was determined to be ineffective due to changing patient contact information and availability to be able to continue data collection post intervention. The 12-month duration of the study, however, exceeds that of many previous trials.

Fourth, this study used a quasi-experimental design with alternating assignment rather than true randomization. The selection of participants was based on a sequential allocation process where every other patient was offered participation in the DCM intervention. The nonrandom assignment process introduces selection bias. Patients who chose to participate in the DCM project may differ systematically from those who declined to do so, particularly in terms of motivation, technological aptitude, or other unmeasured characteristics that could influence treatment outcomes. Self-selection into the experimental group could mean that more motivated or tech-savvy patients were overrepresented in the experimental group, which could impact the generalizability of results. However, we conducted postmatching analyses to reduce observable confounding, and the statistically significant differences observed in primary outcomes suggest that the intervention still had a meaningful impact despite this limitation.

While our PSM approach attempted to balance observed characteristics between groups, it cannot account for unmeasured confounders that might have influenced both group assignment and outcomes. Although we conducted sensitivity analyses to assess the robustness of our findings to unmeasured confounding, selection bias remains an inherent limitation of our design. At the same time, anecdotally, it does not appear that technology was a limiting feature for those opting out. In addition, the variability observed within the appointment and urine rates suggests that individual differences, including socioeconomic status, comorbid conditions, and personal motivation, may affect treatment outcomes. Future research should explore these contextual factors to identify and address potential barriers to successful implementation and should use randomized controlled designs when feasible.

Finally, while our PSM successfully balanced most covariates between treatment and control groups, a moderate imbalance in gender (SMD=0.23) persisted after matching. This exceeds our predetermined threshold of 0.2 and represents a potential limitation. The potential influence of this residual gender imbalance on our results cannot be fully ruled out and should be explored in future studies with larger sample sizes that would enable gender-stratified analyses. Future studies should use more stringent matching algorithms or consider exact matching on gender to eliminate this potential source of bias.

### Conclusions

This article supports the validity of DCM as an intervention to improve treatment adherence and outcomes for people with SUD. For the DCM group, urine test abstinence and appointment attendance were significantly higher than those in treat-as-usual recipients. These results suggest that technology-enabled interventions can improve patient compliance and engagement.

The improvements in both abstinence and appointment attendance suggest that DCM could be a cost-effective addition to SUD treatment, especially considering associated potential reductions in health care utilization, emergency services, and societal costs associated with SUDs. DCM additionally allows for greater accessibility while also removing administrative overhead due to automation. As cell phone usage continues to grow, potential equity concerns should be diminished.

The findings could provide additional support for enacting policies that reimburse DCM as a viable medical protocol that improves treatment compliance and outcomes. Our retention and abstinence outcomes support the inclusion of DCM in emerging value-based payment models. Currently, most state Medicaid programs do not have specific billing codes for CM. However, DynamiCare platform’s verification features address a key concern in current reimbursement policies by providing robust documentation of incentive distribution and behavioral verification, directly meeting Medicaid’s increasing requirements for service documentation and accountability. At the same time, we recommend that state and federal regulatory bodies establish clear guidelines for DCM implementation that address privacy concerns and verification standards.

## Supplementary material

10.2196/73617 Multimedia Appendix 1 Rosenbaum bounds sensitivity analyses on urine test and appointment rates.

## Abbreviations

CM: contingency management
DCM: digital contingency management
HIPAA: Health Insurance Portability and Accountability Act
IRB: institutional review board
PSM: propensity score matching
SMD: standardized mean difference
SUD: substance use disorder
