# Supplementary material for: Digital Contingency Management for Substance Use Disorder Treatment: 12-Month Quasi-Experimental Design
Source: JMIR Ment Health. 2025 Sep 2;12:e73617. doi: 10.2196/73617 (PMC12404579; doi:10.2196/73617)
Supplement: Multimedia Appendix 1 [file mental-v12-e73617-s001.docx]

Table S1. Rosenbaum bounds sensitivity analysis on urine test rate.

| Gamma | sig+ | sig- | t-hat+ | t-hat- | CI+ | CI- |
| --- | --- | --- | --- | --- | --- | --- |
| 1 | 0 | 0 | 0.90 | 0.90 | 0.88 | 0.91 |
| 1.2 | 0 | 0 | 0.89 | 0.91 | 0.87 | 0.92 |
| 1.3 | 0 | 0 | 0.89 | 0.91 | 0.87 | 0.93 |
| 1.4 | 0 | 0 | 0.88 | 0.91 | 0.86 | 0.93 |
| 1.5 | 0 | 0 | 0.88 | 0.91 | 0.86 | 0.94 |
| 1.6 | 0 | 0 | 0.87 | 0.92 | 0.85 | 0.94 |
| 1.7 | 0 | 0 | 0.87 | 0.92 | 0.85 | 0.94 |
| 1.8 | 0 | 0 | 0.86 | 0.93 | 0.84 | 0.94 |
| 1.9 | 0 | 0 | 0.86 | 0.93 | 0.84 | 0.95 |
| 2 | 0 | 0 | 0.86 | 0.93 | 0.84 | 0.95 |

Table S2. Rosenbaum bounds sensitivity analysis on appt rate.

| Gamma | sig+ | sig- | t-hat+ | t-hat- | CI+ | CI- |
| --- | --- | --- | --- | --- | --- | --- |
| 1 | 0 | 0 | 0.67 | 0.67 | 0.63 | 0.69 |
| 1.2 | 0 | 0 | 0.64 | 0.68 | 0.61 | 0.71 |
| 1.3 | 0 | 0 | 0.64 | 0.69 | 0.60 | 0.72 |
| 1.4 | 0 | 0 | 0.63 | 0.70 | 0.59 | 0.73 |
| 1.5 | 0 | 0 | 0.62 | 0.71 | 0.58 | 0.74 |
| 1.6 | 0 | 0 | 0.61 | 0.72 | 0.58 | 0.75 |
| 1.7 | 0 | 0 | 0.61 | 0.72 | 0.57 | 0.75 |
| 1.8 | 0 | 0 | 0.60 | 0.73 | 0.56 | 0.76 |
| 1.9 | 0 | 0 | 0.59 | 0.73 | 0.56 | 0.76 |
| 2 | 0 | 0 | 0.58 | 0.74 | 0.55 | 0.77 |

The treatment effect for this outcome is highly robust to potential unmeasured confounding.

Even with significant hidden bias (Γ=2\Gamma = 2Γ=2), the treatment effect remains statistically significant and meaningful.
